# Supplementary material for: The prognostic significance of lymph nodes in patients with pT1c33N0M0 non-small cell lung cancer: a retrospective study
Source: PeerJ. 2024 Jan 31;12:e16866. doi: 10.7717/peerj.16866 (PMC10838084; doi:10.7717/peerj.16866)
Supplement: Supplemental Information 2 [file peerj-12-16866-s002.docx]

STROBE Statement—checklist of items that should be included in reports of observational studies

|  | Item No. | Recommendation | Page  No. | Relevant text from manuscript |
| --- | --- | --- | --- | --- |
| **Title and abstract** | 1 | 1. Indicate the study’s design with a commonly used term in the title or the abstract | 1 | The objective of this study was to appraise the prognostic impact of lymph nodes in patients diagnosed with pT1c33N0M0 non-small cell lung cancer (NSCLC) and to delve into the prognostic significance of lymph nodes located at the N1 lymph node station in this patient cohort. |
|  |  | (*b*) Provide in the abstract an informative and balanced summary of what was done and what was found | 1 | A retrospective analysis of clinical data was conducted for 255 patients diagnosed with pT1c33N0M0 NSCLC. Lymph nodes were tabulated and categorized into three groups (0-10 nodes, 11-16 nodes, >16 nodes). Clinical data among these three groups of pT1c33N0M0 NSCLC patients were compared. We conducted both univariate and multivariate analyses to pinpoint the factors that impact the prognosis of patients with pT1c33N0M0 non-small cell lung cancer (NSCLC). Additionally, we employed Receiver Operating Characteristic (ROC) curve analysis to pinpoint the optimal lymph node criteria at the N1 station for prognostic prediction in pT1c33N0M0 NSCLC patients. The study found that lymph nodes serve as an independent prognostic factor for pT1c33N0M0 NSCLC patients. Detecting at least 3 or more lymph nodes at the N1 station is associated with a more favourable prognosis in pT1c33N0M0 NSCLC patients. |
| Introduction | | | |  |
| Background/rationale | 2 | Explain the scientific background and rationale for the investigation being reported | 1-2 | Current clinical management of NSCLC often involves surgical removal of the affected lung lobe followed by comprehensive mediastinal lymph node dissection. For patients with lymph node involvement, adjuvant chemotherapy is usually necessary to reduce the risk of recurrence. The accuracy of lymph node detection is pivotal in tailoring treatment strategies . Lymph nodes play a significant role in pathological staging and prognosis assessment, as examining an appropriate number of lymph nodes enhances the reliability of staging and the precision of prognosis evaluation However, there is no established consensus on the optimal number of lymph nodes to be removed during surgery for pT1c33N0M0 NSCLC patients. Furthermore, most research has concentrated on the number of mediastinal lymph nodes, with limited investigation into lymph nodes at the N1 lymph nodes station (stations 10-14). |
| Objectives | 3 | State specific objectives, including any prespecified hypotheses | 2 | Henceforth, this study conducts a retrospective examination of clinical data pertaining to 255 patients diagnosed with pT1c33N0M0 NSCLC who underwent curative lung cancer surgery. The primary goal is to investigate the correlation between various lymph nodes and the most advantageous quantity of N1 station lymph node dissections concerning the prognosis of pT1c33N0M0 NSCLC patients, with the ultimate aim of furnishing dependable guidance for clinical treatment decisions. |
| Methods | | | |  |
| Study design | 4 | Present key elements of study design early in the paper | 2 | We undertook a retrospective examination of 255 individuals diagnosed with pT1c33N0M0 NSCLC . |
| Setting | 5 | Describe the setting, locations, and relevant dates, including periods of recruitment, exposure, follow-up, and data collection | 2 | We conducted a two years and a half (January 2020 to July 2022) retrospective analysis in Beijing Chest Hospital, Capital Medical University. |
| Participants | 6 | (*a*) *Cohort study*—Give the eligibility criteria, and the sources and methods of selection of participants. Describe methods of follow-up  *Case-control study*—Give the eligibility criteria, and the sources and methods of case ascertainment and control selection. Give the rationale for the choice of cases and controls  *Cross-sectional study*—Give the eligibility criteria, and the sources and methods of selection of participants | 2 | The inclusion criteria of patients in this retrospective study were: (1) histopathologically confirmed NSCLC with a pathological stage of pT1c33N0M0, (2) patients who underwent systematic mediastinal lymph node dissection, and (3) availability of complete medical records. Exclusion criteria included: (1) patients with severe cardiopulmonary insufficiency, (2) preoperative neoadjuvant therapy, (3) sublobar resection or the absence of systematic lymph node dissection, (4) intraoperative discovery of thoracic or distant metastasis, and (5) incomplete medical records. |
|  |  | (*b*) *Cohort study*—For matched studies, give matching criteria and number of exposed and unexposed  *Case-control study*—For matched studies, give matching criteria and the number of controls per case |  |  |
| Variables | 7 | Clearly define all outcomes, exposures, predictors, potential confounders, and effect modifiers. Give diagnostic criteria, if applicable | 2 | Within the group of 255 patients diagnosed with pT1c33N0M0 NSCLC, 169 were male, and 86 were female, spanning an age range of 33 to 81 years. A collective count of 3902 lymph nodes was identified, equating to an average of 15.3 lymph nodes per patient. Lymph nodes were divided into three groups based on quartiles: <10 nodes, 11-16 nodes, and >16 nodes, as there were no specific grouping criteria for lymph nodes. |
| Data sources/ measurement | 8* | For each variable of interest, give sources of data and details of methods of assessment (measurement). Describe comparability of assessment methods if there is more than one group |  | *N/A* |
| Bias | 9 | Describe any efforts to address potential sources of bias |  | N/A |
| Study size | 10 | Explain how the study size was arrived at |  | Through the electronic medical records, 255 patients who diagnosed with pT1c33N0M0 NSCLC in our hospital from January 2020 to July 2022 were selected. |

Continued on next page

| Quantitative variables | 11 | Explain how quantitative variables were handled in the analyses. If applicable, describe which groupings were chosen and why |  | N/A |
| --- | --- | --- | --- | --- |
| Statistical methods | 12 | (*a*) Describe all statistical methods, including those used to control for confounding | 3 | Data were analyzed using SPSS 25.0 software. Chi-square tests were used to compare clinical and pathological data among multiple patient groups. Kaplan-Meier analysis was employed to calculate survival rates, and differences between groups were assessed using the Log-rank test, including trend tests. Variables with *P* < 0.3 in univariate analysis were included in a Cox proportional hazards model for multivariate survival analysis. The receiver operating characteristic (ROC) curve analysis was employed to establish the ideal cutoff value for prognostic predictions in pT1c33N0M0 NSCLC patients, focusing on the N1 station. Statistically significant distinctions were recognized (*P* < 0.3). |
|  |  | (*b*) Describe any methods used to examine subgroups and interactions |  |  |
|  |  | (*c*) Explain how missing data were addressed |  |  |
|  |  | (*d*) *Cohort study*—If applicable, explain how loss to follow-up was addressed  *Case-control study*—If applicable, explain how matching of cases and controls was addressed  *Cross-sectional study*—If applicable, describe analytical methods taking account of sampling strategy |  |  |
|  |  | (*e*) Describe any sensitivity analyses |  |  |
| Results | | | | |
| Participants | 13* | (a) Report numbers of individuals at each stage of study—eg numbers potentially eligible, examined for eligibility, confirmed eligible, included in the study, completing follow-up, and analysed | 3 | The original investigation of our hospital's electronic medical system showed 308 individuals diagnosed with pT1c33N0M0 NSCLC who received treatment from January 2020 to July 2022. Fifty-three patients meeting the exclusion criteria were removed from the study. |
|  |  | (b) Give reasons for non-participation at each stage | 3 | After excluding 17 patients complicated with severe chronic physical diseases and 36 cases with incomplete data, 255 patients were eligible for this study. |
|  |  | (c) Consider use of a flow diagram |  |  |
| Descriptive data | 14* | (a) Give characteristics of study participants (eg demographic, clinical, social) and information on exposures and potential confounders |  |  |
|  |  | (b) Indicate number of participants with missing data for each variable of interest |  |  |
|  |  | (c) *Cohort study*—Summarise follow-up time (eg, average and total amount) |  |  |
| Outcome data | 15* | *Cohort study*—Report numbers of outcome events or summary measures over time |  |  |
|  |  | *Case-control study—*Report numbers in each exposure category, or summary measures of exposure |  |  |
|  |  | *Cross-sectional study—*Report numbers of outcome events or summary measures |  |  |
| Main results | 16 | (*a*) Give unadjusted estimates and, if applicable, confounder-adjusted estimates and their precision (eg, 95% confidence interval). Make clear which confounders were adjusted for and why they were included | 3 | The outcomes of the univariate analysis revealed significant associations between the 5-year survival rate of pT1c33N0M0 NSCLC patients and variables such as tumour maximum diameter, T stage, and lymph nodes (*P* < 0.05). Those variables with P-values less than 0.3 in the univariate analysis were subsequently included in a Cox regression model for multivariate analysis. |
|  |  | (*b*) Report category boundaries when continuous variables were categorized |  |  |
|  |  | (*c*) If relevant, consider translating estimates of relative risk into absolute risk for a meaningful time period |  |  |

Continued on next page

| Other analyses | 17 | Report other analyses done—eg analyses of subgroups and interactions, and sensitivity analyses |  | N/A |
| --- | --- | --- | --- | --- |
| Discussion | | | | |
| Key results | 18 | Summarise key results with reference to study objectives | 3-4 | The results from this multivariate analysis indicated that tumour maximum diameter, T stage, and lymph nodes remained as independent factors significantly influencing the 5-year survival rate of pT1c33N0M0 NSCLC patients; the results derived from the multivariate analysis demonstrated that tumor maximum diameter, T stage, and lymph nodes had independent impacts on the median survival time of pT1c33N0M0 NSCLC patients, with statistical significance; the ROC curve analysis revealed an area under the curve (AUC) of 0.6982 for predicting the 5-year survival of pT1c33N0M0 NSCLC patients based on the number of N1 station lymph nodes detected. The maximum Youden index, approximately 0.316, occurred when the number of N1 station detected was 2.7 nodes. |
| Limitations | 19 | Discuss limitations of the study, taking into account sources of potential bias or imprecision. Discuss both direction and magnitude of any potential bias | 5 | This study is a single-center retrospective analysis with certain case selection bias and a relatively small sample size. Moreover, the study did not conduct further analysis on specific T stages, which limits the generalizability of the results. Future research should consider conducting multicenter studies with larger samples and a multidimensional approach to further validate the impact of lymph node detection on the prognosis of pT1c33N0M0 NSCLC patients, ultimately aiming to improve postoperative survival rates. |
| Interpretation | 20 | Give a cautious overall interpretation of results considering objectives, limitations, multiplicity of analyses, results from similar studies, and other relevant evidence | 5 | The quantity of identified lymph nodes stands as an independent prognostic determinant for individuals diagnosed with pT1c33N0M0 NSCLC. A minimum of three or more lymph nodes should be detected in the N1 station for better prognosis in these patients. |
| Generalisability | 21 | Discuss the generalisability (external validity) of the study results | 5 | This approach leads to more accurate pathological staging after curative lung cancer surgery, which, in turn, provides patients with a better prognosis . |
| Other information | |  | | |
| Funding | 22 | Give the source of funding and the role of the funders for the present study and, if applicable, for the original study on which the present article is based |  | N/A |

*Give information separately for cases and controls in case-control studies and, if applicable, for exposed and unexposed groups in cohort and cross-sectional studies.

**Note:** An Explanation and Elaboration article discusses each checklist item and gives methodological background and published examples of transparent reporting. The STROBE checklist is best used in conjunction with this article (freely available on the Web sites of PLoS Medicine at http://www.plosmedicine.org/, Annals of Internal Medicine at http://www.annals.org/, and Epidemiology at http://www.epidem.com/). Information on the STROBE Initiative is available at www.strobe-statement.org.
